# An audit of prescribing pattern of anti-epileptic drugs with its effect on therapeutic drug levels and seizure control at a tertiary care public hospital

**DOI:** 10.4314/gmj.v57i3.5

**Published:** 2023-09

**Authors:** Renuka Munshi, Chaitali Pilliwar, Miteshkumar Maurya

**Affiliations:** Department of Clinical Pharmacology, Topiwala National Medical College & BYL Nair Charitable Hospital, Mumbai Central, Mumbai-400008, Maharashtra, India

**Keywords:** therapeutic drug monitoring, epilepsy, breakthrough seizure, anti-seizure medications

## Abstract

**Objective:**

The study objective was to evaluate the prescription pattern and use of anti-seizure medications (ASMs) in patients with a seizure disorder and to evaluate if a change in the ASM dose had a beneficial effect on seizure control, observed through Therapeutic Drug Monitoring [TDM] level of ASMs.

**Methods:**

Details of anti-seizure medications with their therapeutic levels in the blood of patients with seizure disorder were analysed.

**Design:**

Hospital-based retrospective analysis of patient case records

**Settings:**

Therapeutic Drug Monitoring OPD of a tertiary care public teaching hospital

**Participants:**

Case records of 918 patients with seizure disorder from 2016-2021

**Results:**

Data of men (53%) and women (47%) aged between 18-75 years was assessed About 62% (566/918) of patients were on levetiracetam, the most frequently prescribed anti-seizure medication. Whenever the ASMs dose was increased or decreased based on TDM levels, it was associated with a significant increase in the frequency of break-through seizures [OR- 5 (95% CI: 1.28-19.46)]. However, significant seizure control was observed when the patients were on the same maintenance dose of the anti-seizure medication [OR- 0.2 (95% CI: 0.06-0.63)]. Whenever an additional new anti-epileptic drug was prescribed or removed from the pre-existing anti-epileptic medications, it did not significantly impact seizure control.

**Conclusion:**

Individualising drug therapy and therapeutic drug monitoring for each patient, along with patient factors such as medication compliance, concomitant drug and disease history, and pharmacogenetic assessment, should be the ideal practice in patients with seizures for better seizure control.

**Funding:**

None declared

## Introduction

Therapeutic drug monitoring (TDM) is generally defined as the clinical laboratory measurement of a chemical parameter that, with appropriate medical interpretation, will directly influence drug prescribing procedures.^1^ TDM helps individualise drug dosage by maintaining plasma/serum drug concentrations within a targeted therapeutic range (TR). Therapeutic drug monitoring is vital when measuring drugs with a narrow therapeutic range. It can be done for indications such as evaluating drug efficacy, compliance with medications, checking drug interactions, avoiding toxicity due to overdose, and monitoring drug levels following initiation or when planning to stop the therapy.^2^ An essential element of TDM is the provision of accurate and clinically relevant reference intervals. Individuals have different pharmacokinetics and drug responses, particularly in the case of combined therapies, which raises the challenge of universal TDM targets. An essential element of TDM is providing an accurate and clinically relevant reference range. The TDM reference range needs to relate to clinical outcomes in efficacy and toxicity.^3^ The meaning of reference range is largely misunderstood in TDM of anti-seizure medications.

The reference range is a range of drug concentrations quoted by a laboratory. It defines a lower limit below which a therapeutic response is relatively unlikely and an upper limit above which toxicity is relatively likely.

It should be understood that it is important to consider patient-related factors (e.g., diarrhoea, chronic liver or kidney disease, drug-drug interactions, patient compliance, hypoalbuminemia) as well that can affect the plasma or serum anti-seizure medication (ASM) concentration (TDM levels) and optimising correct drug dose becomes important for better seizure control. Hence, the patient's clinical status should be considered while adjusting the dose to achieve the target plasma/serum drug concentration.^4^ The “individual therapeutic concentration/range” concept should be the ideal practice for ASM therapy, which is the concentration associated with an optimum response in an individual patient.^5^ A major advantage of this approach is that it does not rely on fixed “reference ranges” and can be applied to all ASMs regardless of whether or not “reference ranges” have been clearly defined. To further strengthen our understanding and application of individualised drug therapy, we have reviewed the TDM data of patients with epilepsy visiting a public tertiary care hospital.

The study aimed to evaluate the use of different types of anti-seizure medications (ASMs) in patients diagnosed with epilepsy at the Therapeutic Drug Monitoring (TDM) OPD at a tertiary care hospital and to evaluate the effect of change of ASM doses on serum drug level and seizure control.

## Methods

The study was conducted retrospectively using patient records and conducted in compliance with Indian Good Clinical Practice (2001) guidelines and Declaration of Helsinki (2013) principles. The study was accorded an exemption from ethics review. This study reviewed the data of epileptic patients who visited the TDM OPD at our tertiary care referral hospital, Topiwala National Medical College and BYL Nair Charitable Hospital, Mumbai, from January 2016 to February 2021. Only those patient records with idiopathic causes of seizures were considered for data collection and evaluation.

The Case Record Form (CRF) was synthesised to capture demographic details of the patients, seizure details in the past six months that included the number of episodes of breakthrough seizures, any specific or routine indication for doing TDM, concomitant drugs and the number of anti-epileptic drugs, dose alterations (increase or decrease) done in past six months. TDM levels of various anti-seizure medications were also collected to understand if they were within the therapeutic range and if there was any association with the breakthrough seizures. High-Performance Liquid Chromatography (HPLC), available at our Centre for patient care services, was used to monitor the concentrations of the anti-seizure medications in serum.

Although the serum concentrations for many drugs peak 1 to 2 hours after an oral dose is administered, factors such as slow or delayed absorption can significantly delay the time peak serum concentrations are attained. We usually perform therapeutic drug monitoring of anti-seizure medications once they have achieved steady state levels, i.e., after 5½ half-lives of the respective drugs, to obtain drug trough levels in serum samples. The outcome measures were to assess the prescription pattern of anti-seizure medications at single and multiple OPD visits, different combinations of ASMs prescribed and indications for TDM to assess any association between increase/ decrease in drug dose with serum drug levels and the number of breakthrough seizure episodes.

### Statistical analysis

The data from the TDM case record form related to disease information, seizure episodes, prescription of drugs and change of doses or drugs were analysed using descriptive and inferential statistics using the statistical tool IBM SPSS version 23. All results were analysed for significance at a 5% confidence level. The Crude odds ratio was used to establish an association between a change in anti-seizure medications dose exposure and a decrease in seizure episodes as an outcome with a 95% Confidence interval.

## Results

**Figure 1:**
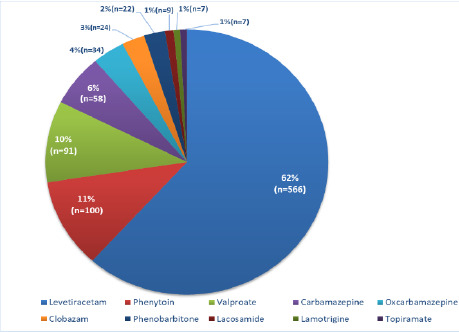
Single OPD Visit: Proportion of patients prescribed with various ASMs (2016-2021) [N=918 patients]

From the years 2016 to 2021, among the anti-seizure medications prescribed, levetiracetam was the most commonly prescribed anti-seizure medication, given in 566 patients (62%), followed by phenytoin, sodium valproate, carbamazepine, oxcarbazepine, clobazam and phenobarbitone. The newer anti-seizure medications, such as lacosamide (9 patients), lamotrigine and topiramate (7 patients each), were less frequently prescribed. For details, refer to Figure 1.

For the review, we rearranged the TDM data in two sets. One set of TDM data contains data on patients who visited only once (single visit) during the study period (2016-2021), and the second set contains data on patients who visited more than once (multiple visits) during the study period. The data set for multiple visits intended to analyse and establish a relationship between drug and drug dose changes and two or more subsequent TDM levels of anti-seizure medications.

### Assessment of prescription pattern of anti-seizure medications at a single visit

Of the 644 patients out of the total 701 patients who paid a single visit to the OPD, levetiracetam remained the most prescribed anti-seizure medication, with 349 patients (54.19%) being prescribed as single drug therapy, followed by phenytoin, sodium valproate, carbamazepine, oxcarbazepine, clobazam and phenobarbitone. The number of patients taking anti-seizure medications increased from the year 2016 onwards, the exception being the years 2020 and 2021 owing to the Covid-19 pandemic and lockdown across India.

**Figure 2:**
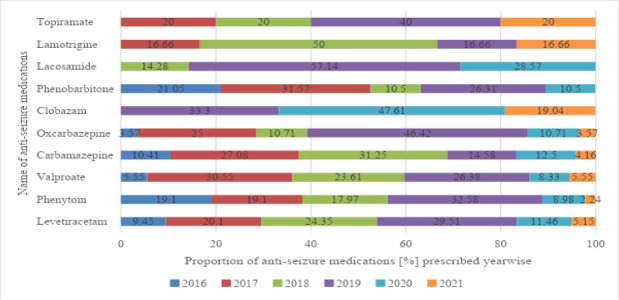
Year-wise prescribing data of anti-seizure medications (single OPD visit)

The trend of prescribing newer drugs like topiramate, lamotrigine, and lacosamide started in recent years. No patient was prescribed these newer drugs in 2016, while only one was prescribed topiramate and lamotrigine in 2017. The details of year-wise prescribing data for anti-seizure medications are represented in Figure 2.

Among the different combinations of anti-seizure medications prescribed in patients giving a single visit to TDM OPD, the combination of phenytoin and levetiracetam was the most commonly prescribed drug combination (55 patients), followed by sodium valproate and levetiracetam combination (33 patients) and carbamazepine and levetiracetam (28 patients).

### Assessment of prescription pattern of anti-seizure medications at multiple visits

**Figure 3:**
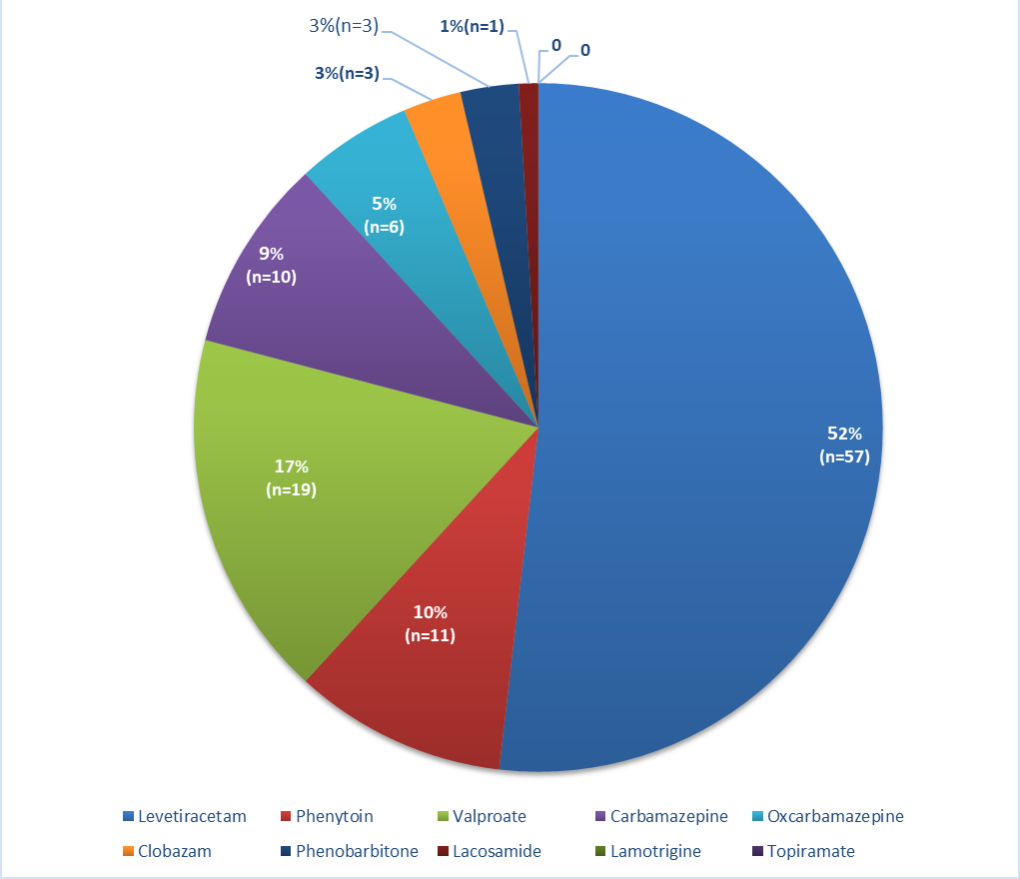
Multiple OPD Visits: Number of ASMs prescribed (2016-2021) **Abbreviations:** Leve- Levetiracetam, Val-Valproate, Cbz- carbamazepine, Phy- Phenytoin, Cloba- Clobazam, Oxcbz- Oxcarbazepine, Laco- Lacosamide, Lamo-Lamotrigine, Topi- Topiramate, Pbt- Phenobarbitone

Among the 57 patients who visited the TDM OPD more than once from 2016 to 2021, levetiracetam was the most prescribed anti-seizure medication, with all 57 patients being prescribed the drug *per se* or in combination with other ASMs. Sodium valproate was the second most commonly prescribed drug, followed by phenytoin, carbamazepine, oxcarbazepine, clobazam, phenobarbitone and topiramate (Figure 3).

Further analysis of patients with more than one seizure was done to establish a relationship between a change in drug, dose and two or more subsequent TDM levels of anti-seizure medications.

The TDM data from the data sets included 57 adult patients, with a total of 148 collected samples available for analysis. From the various ASM combinations in 57 patients, levetiracetam as a single therapy was prescribed the maximum, 15 times, followed by the combination of levetiracetam and sodium valproate, levetiracetam with carbamazepine and levetiracetam with phenytoin. The different combinations of the ASMs are represented in Table 1.

**Table 1:** Different ASM Combinations (multiple visits)

ASM Drug Combinations [N= 57]	Number of prescriptions from the year 2016 to 2021 n [%]
Leve	15(26.31)
Leve + Val	9(15.78)
Leve + Cbz	6(10.52)
Leve + Phy	7(12.28)
Leve + Oxcbz+ Topi	3(5.26)
Leve + Pbt	1(1.75)
Leve + Val + Oxcbz + Phy	1(1.75)
Leve + Cbz + Oxcbz	1(1.75)
Leve + Val + Oxcbz	1(1.75)
Leve + Val + Laco	1(1.75)
Leve + Cbz + Laco	1(1.75)
Leve + Val + Lamo	1(1.75)
Leve + Phy + Cloba	1(1.75)
Leve + Val + Cloba	1(1.75)
Leve + Topi+ Oxcbz + Cloba	1(1.75)

In 2021, there were only single visits till March 2021. Of the 57 patients who visited TDM OPD more than once, their anti-seizure medications combinations, indication for TDM, TDM levels, the occurrence of BTC, change in drug dose post estimation of TDM levels, and subsequent change in TDM levels were calculated. The indications for therapeutic drug monitoring in patients with epilepsy are described in Table 2.

**Table 2:** Number of patients with epilepsy and their TDM Indications

Indications for TDM [N= 57]	No. of patients n (%)
Breakthrough seizure	39 (68.4)
Non-responders	5 (8.77)
To check compliance	4 (7.01)
Suspected toxicity	4 (7.01)
Following a dose change or initiation of therapy	3 (5.26)
Routine follow up	1 (1.75)
Overdose	1 (1.75)

Most patients (68.4%) visited the OPD following a breakthrough seizure episode. Out of 57 patients, after the first OPD visit, the dose of the ASM was changed in 23 patients, new ASMs were added in 10 patients, ASMs were deleted in 13 patients, and no change in ASM dose was seen in 19 patients. The increase/decrease in dose and addition/reduction in the number of drugs was in the same patient in some instances. The dose of anti-seizure medications was increased in 20 patients, and levetiracetam was increased in 18. After increasing the dose of ASM, 17 patients experienced a seizure episode (BTC) till the next visit, i.e., visit 2, while only three patients had no seizure episode before visit 2 (Table 3).

**Table 3:** Association between change in dose and occurrence of breakthrough convulsion (BTC) post-dose change

Anti-seizure medications Dose modifications	Total Number of patients [N]	Number of patients followed with BTC n [%]	Number of patients followed with No BTC n [%]	Crude Odds ratio with 95% CI	p Value
Change in ASM dose	23	20 (86.95)	3 (13.04)	5(1.28-19.46)	0.03*
Increased dose	20	17(85)	3(15)	3.8(0.96-14.71)	0.08
Decreased dose	3	3(100)	0 (0)	0.54(0.17-73.52)	0.54
No change in the dose	19	8 (42.11)	11(57.89)	0.2(0.06-0.63)	0.01*
Addition of new ASM	10	8(80)	2(20)	2.11(0.40-10.95)	0.59
Reducing the number of ASMs	13	8 (61.53)	5(38.46)	0.71(0.20-2.51)	0.84

Using 2 x 2 Chi-square statistics, *p<0.05 for statistical significance

During further evaluation, it was observed that 14 out of 17 patients (who experienced a BTC despite the increase in dose) had drug levels within the therapeutic range (TR); two patients had below TR and one above TR (refer to Table 4).

**Table 4:** Association of breakthrough convulsion [BTC] with drug levels with dose adjustment (Increase)

	Number of patients [N]	Within TR n [%]	Below TR n [%]	Above TR n [%]
**BTC**	17	14(82.35)	2(11.76)	1(5.88)
**No BTC**	3	2(66.66)	0(0)	1(33.33)

While the TDM level of nine patients improved on increasing the dose, seven patients' TDM levels remained the same as earlier (i.e., within range), and three patients' TDM levels increased (above range). In contrast, one patient's TDM level deteriorated. Of the 57 patients, there was no change in the dose of anti-seizure medications in 19 patients after the first OPD visit.

In eight patients with a seizure episode, levetiracetam was within therapeutic range in six patients, while one patient had below TR and one above TR. In 11 out of 19 patients who did not experience a BTC, levetiracetam TDM level was within the therapeutic range in ten patients, while it was below the therapeutic range in only one patient (refer to Table 5).

**Table 5:** Association of breakthrough convulsion [BTC] with drug levels with no dose adjustment

Variable	Number of patients [N]	Within TR n [%]	Below TR n[%]	Above TR n [%]
**BTC**	8	6 (75)	1 (12.50)	1 (12.50)
**No BTC**	11	10 (90.90)	1 (9.09)	0 (0)

The dose of anti-seizure medications was decreased in three patients, wherein levetiracetam was decreased in all three of them. In 10 out of 57 patients, new ASMs were added by the physician post-first visit after observing the TDM levels, with more than one ASM added in a few patients. Carbamazepine was added in five, topiramate in two, sodium valproate in two, phenytoin in three, phenobarbitone in one and oxcarbazepine in one patient. The anti-seizure medications were stopped in 13 patients, where levetiracetam was stopped in four patients, sodium valproate in four, carbamazepine in three, phenytoin and oxcarbazepine in two patients each and topiramate and lamotrigine in one patient each. Overall, 35 of 57 patients had at least one seizure episode in the subsequent visit, 23 had drug levels within the therapeutic range, 10 with drug levels below the therapeutic range and two with drug levels above TR. On the other hand, 22 patients had no seizure episodes in subsequent visits. In contrast, 15 had drug levels within the therapeutic range, five patients with drug levels below the range (these patients were on combined therapy, of which three patients had other drug levels within TR), and one of the 22 patients had drug levels above the therapeutic range.

## Discussion

Of the total 566 epileptic patients, 62% were maintained on the drug levetiracetam, the most commonly prescribed anti-seizure medication in this audit. It has been in the market for the last two decades with pharmacokinetic advantages, including rapid and almost complete absorption, minimal binding to plasma protein, absence of enzyme induction, absence of interactions with other drugs, and partial metabolism outside the liver. It is effective as an adjunctive therapy for refractory partial-onset seizures, primary generalised tonic-clonic seizures, and myoclonic seizures of juvenile myoclonic epilepsy. Levetiracetam is also commonly given with other ASMs as a concomitant medication in patients whose seizures are not controlled by a single ASM.^6^

A study by Moran N.F. et al^7^ evaluated the impact of the use of anti-seizure medications on seizure frequency and severity in 1652 epileptic patients in the United Kingdom.^7^ In this study, the most commonly used ASMs were carbamazepine (37.4%), followed by sodium valproate (35.7%) and phenytoin (29.4%). In comparison, our study revealed levetiracetam (62%) as the most commonly prescribed anti-seizure medication, followed by phenytoin, sodium valproate, and carbamazepine.

Monotherapy was given to 68% of the patients, while in our study, 54.19% were exposed to monotherapy. Patients taking multiple ASMs reported significantly higher levels of adverse effects, worse seizure control, and declining seizure frequency with increasing age, which our study did not assess. The systematic review and meta-analysis performed by Kesselheim AS. *et al* in 2010 concluded no significant association between the generic substitution of branded drugs with generic anti-seizure medications and seizure control.^8^

A 10-year assessment by Ahmed KA *et al* in 9134 seizure-diagnosed infants in US neonatal intensive care units who received an anti-epileptic drug revealed that the phenobarbital [98%] was most commonly used ASM since 2005. However, this exposure declined significantly to 96% between 2013–2014, while phenytoin exposure decreased from 15 to 11%. However, levetiracetam exposure significantly increased 10-fold from 1.4 to 14%, similar to our study findings of drug utilisation. Overall, <1% of infants were exposed to carbamazepine.^9^ Chen S *et al* evaluated the prescription patterns of anti-epileptic drugs in 6024 adult patients in Japan with newly diagnosed focal epilepsy [2006 to 2017], excluding idiopathic seizure cases, unlike our study. The prescription of new ASMs increased significantly, up to 36.8 % of all prescriptions in 2017 compared to 2006. Among new ASMs, prescriptions for levetiracetam increased rapidly and were followed by lamotrigine, similar to our drug utilisation findings.

In contrast, prescriptions for older ASMs, especially valproate, decreased over this same period.^10^ Beghi E. *et al* reviewed both US and UK guidelines and compared the recommendations that revealed US guidelines recommend treatment in newly diagnosed epilepsy with a standard drug or a new drug depending on the individual patient's characteristics. In contrast, the UK guidelines recommend that a new anti-epileptic drug should be considered only if there is no benefit from an older generation of anti-seizure medications or if an old or first-generation drug is contraindicated or if there is a previous negative experience with the same drug, or the patient is a woman of childbearing potential.^11^

Our study had certain limitations. Firstly, we did not consider patient compliance with anti-epileptic medications that can affect the therapeutic levels of the drugs. Moreover, the study was carried out retrospectively with different time points of drug levels being performed. Lastly, there was no evaluation of the concomitant drugs for the drug-drug interaction and patient comorbidities that can affect the drug levels.

## Conclusion

Levetiracetam is the most common anti-seizure medication prescribed by physicians, and there is a need to explore the effect of the new generation of anti-epileptic drugs on seizure control. The change in drug dose did not significantly impact seizure control in patients with idiopathic seizures despite the drug level being within the therapeutic range. However, further prospective study is needed with special consideration to other patient-related factors such as compliance with medications, comorbidities, drug-drug interactions and genotypic association of an individual. Individualised treatment of epileptic patients with optimum drug dose based on drug levels remains the only mainstay for better seizure control.
